# Downregulation of long non-coding RNAs in patients with bipolar disorder

**DOI:** 10.1038/s41598-022-11674-y

**Published:** 2022-05-06

**Authors:** Zahra Maloum, Sahar Ramezani, Mohammad Taheri, Soudeh Ghafouri-Fard, Zeinab Shirvani-Farsani

**Affiliations:** 1 Department of Cell and Molecular Biology, Faculty of Life Sciences and Technology, Shahid Beheshti University, Tehran, Iran; 2 Skull Base Research Center, Loghman Hakim Hospital, Shahid Beheshti University of Medical Sciences, Tehran, Iran; 3 Men’s Health and Reproductive Health Research Center, Shahid Beheshti University of Medical Sciences, Tehran, Iran

**Keywords:** Genetics, Neuroscience, Psychology

## Abstract

The abnormal function of signaling cascades is currently a candidate in the pathophysiology of bipolar disorder (BD). One of the factors involved in activating these signals is oxidative stress. Some long non-coding RNAs (lncRNA) are involved in the oxidative stress. In this study, we compared expression levels of lincRNA-p21, lincRNA-ROR, and lincRNA-PINT in the peripheral blood mononuclear cells (PBMC) from BD patients (n = 50) and healthy individuals (n = 50). Expression levels of lincRNA-p21, lincRNA-ROR, and lincRNA-PINT were significantly reduced in patients with BD compared to controls. In sex-based analyses, down-regulation of these lncRNAs was revealed only in male BD patients compared to male healthy subjects. Also, in BD patients, all three lncRNAs showed a significant pairwise positive correlation in expression level. The area under curve values for lincRNA-p21, lincRNA-ROR, and lincRNA-PINT was 0.66, 0.75, and 0.66, respectively. Thus, the ROC curve analysis showed that lncRNA-ROR might serve as a diagnostic biomarker for distinguishing between BD patients and controls. Altogether, the current study proposes a role for lincRNA-p21, lincRNA-ROR, and lincRNA-PINT in the pathogenesis of bipolar disorder. Moreover, the peripheral expression of these lncRNAs might be useful as potential biomarkers for BD.

## Introduction

Bipolar disorder (BD) is a combination of periodic mania, hypomania, and depression as well as significant sub-syndromic symptoms commonly seen between major mood periods^1^. Moreover, high clinical and familial overlap has been observed between it and other psychiatric illnesses^2^. As with most psychiatric disorders, the exact cause of BD is unknown, but it probably involves a dynamic interaction between genetic susceptibility and several environmental factors^3^. The abnormal function of signaling cascades is currently a candidate in the pathophysiology of BD. Many of these signaling pathways play a role in the cell apoptosis through interfering with integrity of mitochondrial membrane and damaging it. One of the factors involved in activating these signaling pathways and apoptosis is oxidative stress^4^. Several studies have indicated that oxidative stress parameters including nitric oxide levels, antioxidant enzymes, and lipid peroxidation have been altered in patients with BD^5,6^. Rising evidence currently has shown long noncoding RNAs (lncRNAs) have a relevant function in the cellular response to DNA damage and oxidative stress^7,8^. Moreover, they play roles in the neurobiology of BD^9–11^. However, the crosstalk between many lncRNAs and oxidative stress factors in the pathobiology of this mental illness has not been studied. LncRNAs are non-coding RNAs that are longer than 200 nucleotides in length. In general, lncRNAs can affect almost any cellular behavior, from transcription to translation, by various mechanisms, including epigenetic alterations, interacting with miRNAs, proteins, and genetic variants ^12^. In the current research, we assessed expression levels of three lncRNAs, namely long intergenic noncoding RNA (lincRNA)-p21, long intergenic non-protein coding RNA, P53 Induced Transcript (linc-PINT), and lincRNA- regulator of reprogramming (ROR) in peripheral blood of patients with BD and healthy subjects. These lncRNAs were chosen based on their involvement in the pathoetiology of neurological disorders and their participation in the regulation of pathways and cellular processes that are involved in the pathogenesis of BD. We particularly focused on lncRNAs that regulate cellular response to oxidative stress.

lincRNA-p21 can binds to hnRNPK-binding RNAs that suppress downstream genes and regulate p53-dependent apoptosis. Moreover, it can regulate expression of its neighboring gene, p21^13,14^. Expression of lincRNA-p21 activates microglia cells and upregulates expression levels of inflammatory mediators and induces apoptosis in dopaminergic neurons^15^. In addition, expression of lincRNA-p21 has been shown to be enhanced in early stage of Parkinson’s disease and during disease progression with a significantly increased expression in brain stem type Parkinson’s disease^16^. Meanwhile, previous studies have indicated that lincRNA-p21 is linked to oxidative stress via Wnt/β-catenin signaling pathway^15^. Formerly called lincRNA-MKLn1, linc-PINT (p53-induced non-coding transcript) is expressed in all cells and has a p53 binding motif in its promoter^17^. Recent reports highlight that linc-PINT is a neuronal transcript which is upregulated in several brain regions of patients with Alzheimer's, Parkinson's and Huntington's disease^18^. lincRNA-ROR has four exons and contains long terminal repeat (LTR) as well as long and short interspersed nuclear elements^19^. This lncRNA regulates genes involved in the p53 response to oxidative stress and DNA damage. lincRNA-ROR is a negative regulator for P53^20,21^. Several data sources have reported dysregulation of oxidative stress parameters in patients with BD regardless of drug administration or disease phase. In this study, we compared expression levels of three mentioned lncRNAs in the peripheral blood of BD patients and healthy controls to investigate their contribution in the etiology of BD and their potential function as disease biomarker.

## Materials and methods

### Cases and controls subjects

Fifty blood samples from patients with BD and 50 blood samples from healthy controls were collected from Imam Hussein hospital, Behavioral Science Research Center. None of controls had a history of any mental illnesses, infections and cancers. Patients were at euthymic phase at the time of sampling. Female patients were informed about the malformation risk of Carbamazepine and were advised to have safe contraception while taking it. All patients were on Carbamazepine monotherapy.

Blood samples were taken at the same time of the day without fasting. The Diagnostic and Statistical Manual of mental disorders (DSM-5) was used to diagnose patients. Informed consent was signed by all patients and healthy controls or their parents in the case they were under 18 years old. The Ethical Committee Shahid Beheshti University of Medical Sciences approved this study.

### RNA extraction, cDNA synthesis and real-time PCR analysis

Five ml of peripheral blood of patients and controls were transferred to Ethylenediaminetetraacetic acid-containing tubes. For Buffy coat isolation, the blood samples were centrifuged. Total RNA was extracted from the peripheral blood mononuclear cells (PBMC) using the RNAX kit, and then the spectrophotometer was applied for analyzing the RNA quality and quantity.

cDNA was synthesized using Applied Biosystems High-Capacity cDNA Reverse Transcription Kits (PN: 4,375,575). The sequences of primers were used can be found in Table 1; GAPDH was applied in all samples as a reference gene. Quantitative real-time PCR technique with SYBR Green was conducted by an StepOne Plus detection systems (Applied Biosystem/MDS SCIEX, Foster City, CA, USA). The mean of ΔCT for samples was estimated, and the relative gene expression was finely calculated by the 2-ΔΔCt method.

**Table 1:** Primers used in RT-qPCR.

Gene	Forward primer	Reverse primer	Product size (bp)
lincRNA-p21	5ʹGGGGATAAGCACCACTAATG3ʹ	5ʹTGTAGGCAATCACAGAGCAC3ʹ	171
lincRNA-ROR	5ʹ TATAATGAGATACCACCTTA3ʹ	5ʹ AGGAACTGTCATACCGTTTC3ʹ	170
lincRNA-PINT	5ʹAGGAGGGAACGAGGCAGGGA3ʹ	5ʹAGCTCAGATCAGCAAGGCAG3ʹ	129
GAPDH	5ʹCCATGAGAAGTATGACAAC-3ʹ	5ʹGAGTCCTTCCACGATACC-3ʹ	105

### Statistical analysis

All experiments were accomplished at least two times. Statistical analysis was performed using GraphPad Prism 8 (GraphPad Software, Inc., San Diego, CA, USA). We accessed the normal/gaussian distribution of the values by the Shapiro–Wilk test. The t-test was used to examine the differences in genes expression levels between two groups. P-value < 0.05 was considered as significant. Pearson's coefficient of correlation was calculated for assessment of correlation between genes expression and the clinical features. Using this test, we measured the statistical relationship between two continuous variables. The diagnostic value of the genes was determined by the Receiver operator characteristics (ROC) analysis. For this purpose, we plotted the true positive rate against the false positive rate at different thresholds.

### Ethics approval and consent to Participant

All procedures performed in studies involving human participants were in accordance with the ethical standards of the institutional and/or national research committee and with the 1964 Helsinki declaration and its later amendments or comparable ethical standards. Informed consent forms were obtained from all study participants. Informed consent forms were obtained from all study participants. The study protocol was approved by the ethical committee of Shahid Beheshti University of Medical Sciences. All methods were performed in accordance with the relevant guidelines and regulations.

## Results

### Cases and controls

In current study, we recruited 100 subjects, including 50 BD patients and 50 control volunteers. Table 2 shows the general information of the participants.

**Table 2:** Demographic and clinical characteristics of the BD patients and controls.

Clinical characteristics	BD	Controls
No	50	50
Female: male	16:34	19:31
Age (year)	36	34
Age range (year)	17–56	14–52
Disease duration range	1–14	–
Onset age range	15–48	–

### Gene expression analyses in patients with BD compared to the controls

**Figure 1:**
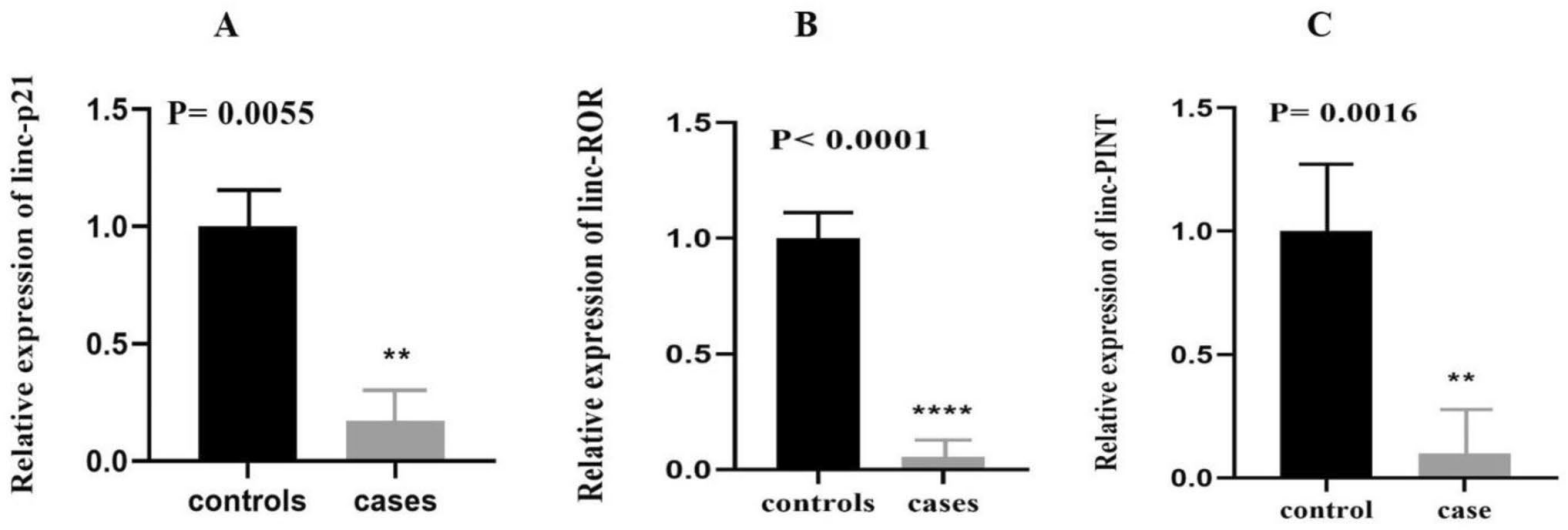
Expression analysis of lncRNAs in the PBMCs. The relative expression (Fold change) of linc-p21 (**A**), lncRNA-ROR (B), and linc-PINT (**C**). Expression of lncRNAs was significantly down-regulated in BD patients compared to controls. Gene expression levels of each sample were normalized relative to GAPDH expression. The relative expression of transcripts was obtained using the formula 2 ^-ΔΔCt^ and t-test.

Our study examined expression of three lncRNAs (lincRNA-p21, lncRNA-ROR, lincRNA-PINT) in BD patients compared with healthy controls. The expression levels of lncRNAs lincRNA-p21 (p = 0.0055), lncRNA-ROR (p = 0.001), lincRNA-PINT (*p* = 0.0016) were statistically significantly different between BD patients and healthy controls (Table 3). Post hoc power calculation using Clincalc online tool (https://clincalc.com/stats/Power.aspx) and expression values of lincRNA-p21 (which had the lowest fold change value in cases versus controls among all lncRNAs) showed that study power was more than 80%. Our study showed that the expression level of lincRNA-p21, lncRNA-ROR, and lincRNA-PINT in BD patients were significantly down-regulated (Fig. 1 A-C). Significant differences in expressions of lincRNA-p21 (P = 0.01), lncRNA-ROR (P = 0.0001), and lincRNA-PINT (P = 0.0012) were seen between male patients and male controls. However, the difference in expressions of these lncRNAs was not statistically significant between female subjects. The relative expression (fold change) of lncRNAs in patients and healthy individuals are presented in Table 3.

**Table 3:** Relative expression of lncRNAs in BD patients and healthy controls.

lncRNAs	Parameters	Total patients (n = 50) total controls (n = 50)	Male patients (n = 34) male controls (n = 31)	Female patients (n = 16) female controls (n = 19)
lincRNA-p21	1/Fold change	5.81	6.36	4.66
	*P*-value	0.005**	0.01*	0.205
lncRNA-ROR	1/Fold change	19.23	28.16	6.10
	*P*-value	0.0001****	0.0001****	0.143
lincRNA-PINT	1/Fold change	10.10	17.85	2.63
	*P*-value	0.0016**	0.0012**	0.42

*significant *p*-value < 0.05, **significant *p*-value < 0.01, ****significant *p*-value < 0.0001.

### Analysis of the relationship between the expression levels of genes

This study calculated the correlation between expression levels of all genes pairs with the Pearson correlation test. The results of this comparison are included in Table 4. We found a significant positive correlation between expression levels of all genes pairs. However, we observed no significant correlation between level of expression of lincRNA-p21, lncRNA-ROR, lincRNA-PINT in BD patients and age, disease duration, and onset age of disease (Table 5).

**Table 4:** Pairwise correlation between expression levels of lncRNAs in cases group.

Correlation	lincRNA-ROR		lincRNA-p21	
lincRNA-PINT	r = 0.756	*P* < 0.0001	r = 0.627	*P* < 0.0001
lincRNA-p21	r = 0.755	*P* < 0.0001

**Table 5:** Correlation analysis between expression levels of lncRNAs and clinical data.

	LincRNA-PINT		lncRNA-ROR		LincRNA-p21	
	R	*P* value	R	P value	R	P value
Patient Age	0.17	0.21	0.006	0.96	0.098	0.49
Age at onset	0.13	0.34	0.53	0.81	0.13	0.36
Disease duration	0.21	0.12	0.12	0.38	0.048	0.73

### ROC curve analysis

**Figure 2:**
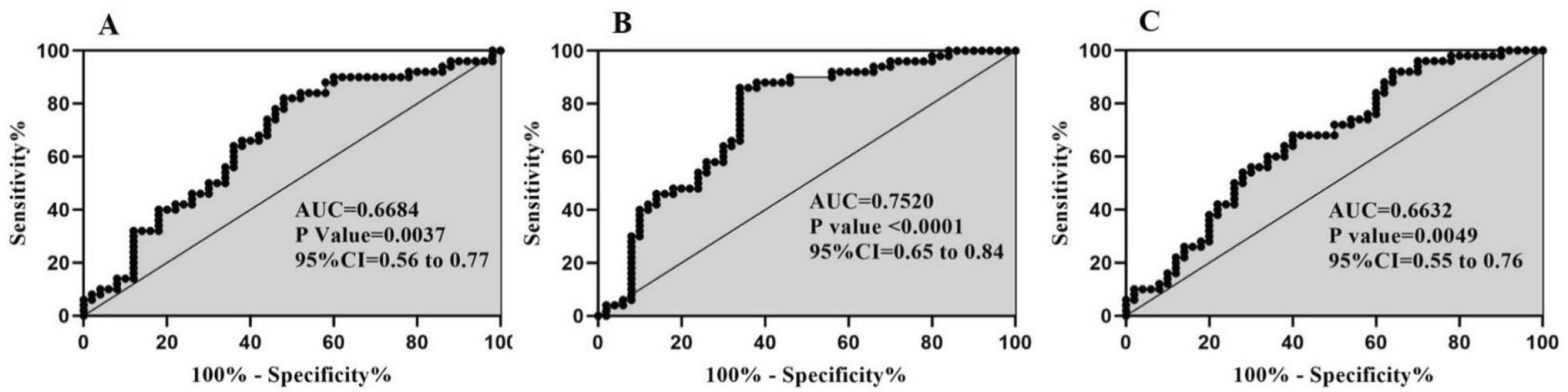
ROC curve analysis of lincRNA-p21 (**A**) lncRNA-ROR (**B**) and lincRNA-PINT (**C**). AUC: area under curve.

The sensitivity and specificity of expression of lincRNA-p21, lncRNA-ROR, lincRNA-PINT as biomarkers was obtained by the Receiver operator characteristics (ROC) analysis. This analysis showed significant results for lincRNA-p21 (AUC = 0.66, P = 0.0037), lncRNA-ROR (AUC = 0.75, P < 0.0001) and lincRNA-PINT (AUC = 0.66, P = 0.0049) (Fig. 2 A-C). The criterion values (cutoff values) of lincRNA-p21, lncRNA-ROR and lincRNA-PINT were 4.30, 5.10 and 1.56 respectively. According to AUC (area under curve) values, all lncRNAs, particularly lncRNA-ROR (sensitivity = 86% and specificity = 73%) can be used as diagnostic biomarkers in bipolar disorder.

## Discussion

The etiology of BD is still unknown, but family studies as well as adoption and twins studies suggest significant contribution of genetic factors^22^. In addition, evidence suggests that altered signaling pathways, including oxidative stress, is one of the general factors involved in the pathogenesis/neurobiology of BD^6^. Reactive oxygen species (ROS) are involved in induction of expression of the p53 tumor suppressor gene and cell aging. p53 has important role in the cellular response to oxidative stress and it is a significant coordinator of oxidative stress and aging^23^. Recently, specific lncRNAs have been identified as p53 targets involved in the regulation of oxidative stress^24^.

Here, we evaluated expression levels of lincRNA-p21, lncRNA-ROR, and lincRNA-PINT that contributed to the oxidative stress pathway, in BD patients and healthy individuals. Our study discovered that expression levels of these lncRNAs are significantly down-regulated in patients with BD compared to controls. Similarly, a comparison of the expression levels of lincRNA-p21, lncRNA-ROR, and lincRNA-PINT in men showed a decrease in expression levels of these lncRNAs in male BD cases compared to healthy males. On the other hand, no significant difference was found in their expression levels between healthy and BD women.

lincRNA-p21 is located near P21 locus on chromosomes 17. This LincRNA was first identified as an intergenic lncRNA regulated by p53 and was reported to have a proapoptotic function in the p53 network. lincRNA-p21 is directly induced and regulated by p53, and it acts as a suppressor of target genes in the p53 pathway^17^. There is a conserved sequence for p53 motifs in the promoter of this lincRNA. Transcriptional suppression by lincRNA-p21 occurs through physical association with hnRNP-K. This interaction is essential for the proper placement of hnRNP-K on the target gene to suppress and regulate the p53 pathway in apoptosis^13,14^. In addition, studies have also shown that knock-down of lincRNA-p21 reduces the p300/p53 interaction, increases the mdm2/P53 interaction, and induced p53 degradation^13^. Xu et al. reported that lincRNA-p21 binds to miR-1277-5p and elevates expression of alpha synuclein (α-Syn), inhibits SH-SY5Y proliferation and induces cell apoptosis^25^. Moreover, lincRNA-p21 binds with miR-181, thus activating microglial cells through the miR-181/PKC-δ pathway^26^. In line with this, lincRNA-p21 suppresses neuronal injury via binding with miR-625 and increasing TRPM2 expression in SH-SY5Y cells^27^.

Another study showed that lincRNA-p21 can bind to CHOP. By inhibiting lincRNA-p21, CHOP protein is more easily ubiquitinated and degraded more rapidly. CHOP is a protein that plays an essential role in stress-dependent apoptosis of the rough endoplasmic reticulum. CHOP can increase the expression of ER (endoplasmic reticulum) reductase genes through production of H2O2 in ER. H2O2 leaks into the cytoplasm and induces inflammation and apoptosis. Increased ROS in ER causes calcium ions to be transported to the cytoplasm, and cytoplasmic ions induce ROS by activating calcium-sensitive kinases, CaMKII, NOX2, and the cytoplasmic subunit NADPH oxidase. These factors stimulate CHOP transcription, resulting in apoptosis. At the same time, CHOP-induced apoptosis can cause cell death by inhibiting P21 cell cycle regulatory protein expression. lincRNA-p21 silencing attenuates cytotoxicity and apoptosis, decreases caspase-3 activity and Bax expression and increases BCL-2 expression. In addition, inhibition of lincRNA-p21 reduces oxidative stress. Decreased levels of TNF-α, IL-IB, and IL-6 are evident with decreased ROS production and increased SOD activity^28^.

lincRNA-ROR regulates expression of genes involved in the p53 response. These genes are responsible for oxidative stress and DNA damage. lincRNA-ROR is a negative regulator for p53. Unlike MDM2 which degrades p53 via the ubiquitin–proteasome pathway, lincRNA-ROR suppresses p53 translation through direct interaction with hnRNP-1^29^. Studies have shown that lincROR acts as a sponge for miR-145. miR-145 plays a regulatory role in the morphogenesis of dendrites, a process that is disrupted in schizophrenia^30^. Also, miR-145 has a functional role in brain tissue. Its overexpression reduces astrocyte damage in ischemic stroke^31^. According to these studies, decreased lincROR expression levels in our study may increase apoptosis and neuronal degradation by increasing the expression of P53 and PETEN. As a result, this lncRNA may be involved in the pathogenesis of BD.

lincRNA-PINT has been shown to be a primary transcript in neurons, and its expression causes a significant increase in the growth of primary nerve cells^18^. Also, increased lincRNA-PINT in the brains of patients with Parkinson’s disease, Alzheimer's disease, and Huntington's disease might be a part of the neuroprotective mechanisms involved in aging-related neurodegenerative processes. These results illustrate the complexity of the role of PINT in the brain, which is neuron-centered, increasing with dendritic growth and advanced nerve damage, and decreasing with age^18^. This process may be related to the interaction of PCR2 which suppresses genes involved in cell death and neuronal cell death in general^32^. In another study, a decrease in PINT expression mediated by an RNAi caused death of N2A and SH-SY5Y cells exposed to oxidative stress, which showed its protective role in neurons^18^.

Gene expression in every part of the brain and even in other tissues may be different. Simchovitz et al. showed that expression of lincRNA-PINT was significantly reduced only in SN samples of patients compared to healthy individuals. This subject indicates the complexity of gene expression in the brain^18^. Since we observed a decrease in expression in lincRNA-PINT in peripheral blood, it can be speculated that this lincRNA may be involved in accelerating the aging of brain cells, thereby contributing to BD.

Our results showed significant reduction of lincRNA-p21, lncRNA-ROR, lincRNA-PINT expression only in male BD patients compared to male controls, which might suggest specific function of these lncRNAs among males or influences of sex hormones/testosterone on expressions of lncRNAs. Alternatively, since the number of females was less than males, this might be due to a power issue.

Meanwhile, pairwise correlations between lncRNAs expressions in patients with BD may indicate similar regulatory functions in pathways important for BD pathogenesis.

In addition, the current study demonstrated that lincRNA-p21, lincRNA-PINT and especially, lncRNA-ROR, may help distinguish BD from healthy individuals as diagnostic biomarkers. However, validation of these data needs a large number of BD samples and controls.

Collectively, our analysis showed that a decrease in blood expression of lincRNA-p21, lncRNA-ROR, and lincRNA-PINT may be linked with an increased risk of BD. Additionally; lncRNA-ROR may serve as a diagnostic biomarker for BD. However, our result needs validation in larger samples of patients. Moreover, we could not exclude the impact of medication on expression of genes. It is necessary to include a group of drug-naïve patients to assess this point.

Finally, development of computational models is a novel strategy for identification of the non-coding RNA biomarkers of human complex diseases in BD. This strategy has been applied in different settings. For instance, miRNA-disease associations have been predicted based on inductive matrix completion^33^. Moreover, several studies have indicated the impact of computational models in identification of novel miRNA-disease associations, which can result in the selection of the most important miRNA-disease pairs for further investigations in experiments^34^. The method of Laplacian Regularized Least Squares for LncRNA-Disease Association has also led to identification of several disease-lncRNA associations^35^. Similar strategies can be applied for detection of biomarkers in BD. Several novel computational models have been used to classify disease-associated lncRNAs on large scales and choose the most suitable disease-associated lncRNAs^36^. It is worth mentioning that construction of systematic functional annotation methods is crucial to enhance the prediction accurateness of computational models and further acceleration of the documentation processes of new lncRNA functions^37^.

## Data Availability

The datasets used and/or analyzed during the current study are available from the corresponding author on reasonable request.
